# Translation, cultural adaptation, and content validity evaluation of a mental health literacy instrument in Bolivia

**DOI:** 10.3389/fpubh.2026.1685333

**Published:** 2026-02-25

**Authors:** Armando Basagoitia, María Teresa Solís-Soto, Martin Romero-Martínez, Jacqueline Elizabeth Alcalde-Rabanal, María Soledad Burrone

**Affiliations:** 1 Instituto Nacional de Salud Pública, Cuernavaca, Mexico; 2 OH TARGET Competence Center, Universidad San Francisco Xavier, Sucre, Bolivia; 3 Instituto de Ciencias de la Salud, Universidad de O'Higgins (UOH), Rancagua, Chile

**Keywords:** health literacy, mental health, psychometrics, surveys and questionnaires, translations

## Abstract

**Background:**

Mental health literacy (MHL)—individuals’ knowledge, attitudes, and beliefs about mental health—plays a key role in promoting public mental health. Accurate MHL measurement helps identify knowledge gaps, reduce stigma, and encourage help-seeking. However, culturally validated instruments remain scarce in low- and middle-income countries, including Bolivia.

**Objectives:**

This study aimed to carry out the translation cultural adaptation and content validity assessment of an instrument designed to comprehensively assess the construct of mental health literacy in the Bolivian urban context.

**Methods:**

A mixed-methods design was used, including forward and backward translation, following best practices for cultural adaptation of patient-reported outcomes. Three instruments were adapted: the Mental Health Literacy Scale (MHLS), O’Connor, 2015; the Perceived Devaluation and Discrimination Scale (PDDS), Link, 1987; and the Mental Health Problem Recognition module (MHPR), Burrone, 2021. Content validation assessment was conducted with eight experts who rated each item’s relevance, comprehensiveness, and comprehensibility using a 4-point Likert scale. Item-level Content Validity Index (I-CVI), modified kappa, Scale-level Content Validity Index (S-CVI), and readability statistics were calculated. This was followed by semi-structured interviews with the same experts to refine items and assess conceptual clarity. A pilot test with 10 participants evaluated comprehension and usability.

**Results:**

The I-CVI scores from the quantitative phase indicated that the majority of items were rated as “good” or “excellent” in terms of relevance (97.14%), comprehensiveness (83.02%), and comprehensibility (94.34%). The obtained S-CVI/UA scores fell well below the recommended threshold for relevance (0.32), comprehensiveness (0.25), and comprehensibility (0.32), indicating a lack of universal agreement among experts. The S-CVI/Ave scores indicated moderate consensus among experts for relevance (0.88), comprehensiveness (0.84), and comprehensibility (0.87). Items that did not achieve optimal I-CVI and modified kappa scores were examined during the qualitative phase. Experts identified minor issues related to terminology and phrasing that could affect clarity. Following minor revisions, all items were retained.

**Conclusion:**

This study presents a culturally adapted, content-validated instrument for assessing MHL in Bolivia. The final version, consisting of 53 items, demonstrates strong content validity and provides a solid foundation for further psychometric evaluation and broader application in Spanish-speaking and Latin American contexts.

## 1 Introduction

Mental health problems are a significant global health issue, affecting millions of individuals worldwide and contributing to a substantial burden on healthcare systems, economies, and societies. According to the World Health Organization (WHO), nearly 1 in 8 people live with a mental health disorder in the world (1). Mental health issues are associated with considerable morbidity and mortality, as well as reduced quality of life (2), this situation only worsens in low- and middle-income countries (LMICs), where mental health problems present unique and exacerbated challenges due to a combination of factors including limited resources, inadequate healthcare infrastructure, and sociocultural barriers (3).

The mental health infrastructure in LMICs frequently suffers from underfunding, with a significant proportion of health budgets allocated to physical health rather than mental health services (4). As a result, mental health services are often integrated into primary healthcare systems or provided in an ad-hoc manner, leading to fragmented and inconsistent care. Furthermore, there is a critical shortage of mental health professionals in many LMICs, compounding the challenges faced by individuals with mental health problems (5). Moreover, socioeconomic factors such as poverty, unemployment, and educational disparities contribute to the prevalence and severity of mental health problems (6), as well as stigma and discrimination against mental health problems, further complicating efforts to provide effective care and support (7). All of these factors influence people’s limited skills to recognize mental health problems in a timely manner, or lead to delay in seeking help to take care of their mental health, which increase the complications and overall impact of these issues on both individual and community health.

To respond to these challenges and limitations, Mental health literacy (MHL) has emerged as “the foundation for mental health care, prevention of mental disorders, and promotion of good mental health”, composed by the “understanding about how to optimize and maintain good mental health, understanding mental disorders and their treatment, decreasing stigma, and enhancing help seeking efficacy” (8, 9). Improving MHL is crucial in addressing the challenges associated with mental health problems (10), particularly in LMICs where mental health literacy is often low, since individuals with a high level of MHL are more likely to recognize early signs of depression or anxiety, seek timely treatment, and adhere to prescribed therapies. Furthermore, improved MHL can help reduce the stigma associated with mental health problems, encouraging individuals to seek help without fear of discrimination.

Mental health literacy is a key strategy for strengthening mental health care at the community level. In this context, primary health care settings represent a strategic platform for its promotion, given the close and continuous interaction between the general population and frontline health personnel (11). This interaction enables the early identification of mental health needs and facilitates targeted, context-sensitive interventions (12). While MHL efforts often focus on the general public, they must also address primary health professionals, who play a decisive role in shaping beliefs, attitudes, and help-seeking behaviors related to mental health. Their position enables them to act as mediators in the reduction of stigma and the promotion of access to care (13), and should have the basic MHL-related competencies to interact effectively with the population at the same basic level. However, evidence reveals that health workers at this level frequently present limited MHL, which can compromise their ability to support patients effectively (14, 15).

Therefore, in order to strengthen health workers competencies, it is essential to assess their MHL systematically. Incorporating MHL assessment into professional development and public health planning contributes to building a workforce equipped to respond to mental health needs in a culturally competent and evidence-informed manner. Ultimately, assessing MHL at a basic level in both target groups—health professionals and the general population—is a necessary first step toward developing strategies aligned with real needs and reducing the mental health treatment gap.

In this sense, several instruments have been developed to assess MHL, each with varying scopes and methodologies. These tools generally aim to evaluate knowledge, attitudes, and behaviors related to mental health (16, 17), usually relying on self-report measures used to gauge an individual’s understanding of mental health issues. However, they might not always account for cultural differences or context-specific factors that can influence mental health literacy, and there remains a substantial gap in the availability of validated instruments in Spanish that could be used in Latin-American countries, since most existing MHL instruments were developed and validated in high-income, English-speaking countries, and their direct use in LMICs raises concerns regarding conceptual equivalence and contextual relevance (8, 18). Thus, validated and culturally adapted instruments are essential for conducting high-quality MHL assessments, particularly in diverse cultural contexts. Standardized tools that have been validated for specific populations ensure that the measures are reliable and accurate in assessing mental health literacy within that context, and cultural adaptation is crucial for ensuring that instruments are relevant and appropriate for the target population.

Although MHL has been extensively researched in Europe and North America, it is still largely unexplored in many low- and middle-income countries, such as Bolivia, a country in Latin America with over 11 million inhabitants by 2024 (19), faces structural challenges that impact mental health care. Its diverse population—comprising over 36 ethnic groups—and high rural residency complicate access to culturally appropriate services (20). Despite notable advances, the country has a low Human Development Index by 2025 (0.675) (21), with 36.3% of the population experiencing moderate poverty, and 11% living in extreme poverty by 2022, and an inequality Gini Index of 0.42 by 2021 (22)—factors strongly associated with mental health disparities and barriers to care (23, 24). While in Bolivia, by 2018 the burden of mental, neurological, substance use disorders, and suicide is significant, accounting for up to 32% of the disease burden in the population aged 20–24 years, and representing up to 33% of the Bolivian Disability-Adjusted Life Years (DALYs) (25), there is limited mental health workforce and insufficient education that contribute to stigma, underdiagnosis, and poor treatment adherence (26, 27). Bolivia’s parliament has pointed out the extremely low allocation of the national health budget to mental health—reportedly less than 0.2% in 2022—the lowest in the Americas, a position it has held since 2015 (28). In parallel, various sectors and organizations have issued repeated calls and advocacy efforts urging the government to take a more active role in strengthening mental health care within the national health system, which currently has no officially updated plan or structure (29).

Latin-American countries present particular cultural beliefs, explanatory models of mental illness, and help-seeking behaviors across countries. While some experiences in adapting and validating mental health-related instruments have been completed (30, 31), these experiences cannot be directly extrapolated to Bolivia, given differences in health system organization, sociocultural contexts, and population characteristics (32). In Bolivia, the absence of culturally adapted and validated MHL instruments constrains the systematic assessment of mental health knowledge, stigma, and help-seeking behaviors among both the general population and primary healthcare professionals. This gap undermines the development of evidence-informed interventions and policies. In such scenario, the cultural adaptation and content validity assessment are essential to ensure that MHL instruments adequately capture locally meaningful dimensions of mental health literacy and avoid construct bias or misinterpretation. By incorporating the local contest into the assessment tools, the instruments can better reflect the realities of the local population and provide more accurate and actionable insights.

Furthermore, content validity assessment is an important step to ensure that the instruments accurately measure what they are intended to assess and also that the instruments are both reliable and valid for the target population. Without this assessment, there is a risk that the results may be misleading or not reflective of the true state of mental health literacy. Beyond its methodological relevance, the availability of a culturally adapted and content-validated MHL instrument has significant implications for advancing Sustainable Development Goal 3 (SDG 3), which aims to ensure healthy lives and promote well-being for all. Mental health is explicitly included within SDG 3, particularly through target 3.4, which emphasizes the promotion of mental well-being and the reduction of premature mortality from non-communicable diseases (33). In this context, a validated MHL instrument provides a crucial evidence base for public health planning, enabling the identification of population-level gaps in mental health knowledge and attitudes, monitoring progress over time, and informing the design of targeted interventions, particularly important in resource-constrained settings such as Bolivia.

In summary, the translation, cultural adaptation, and content validation of an MHL instrument for the urban Bolivian context address a critical methodological and public health gap. By enabling accurate and culturally grounded assessment of mental health literacy among the general population and primary healthcare professionals, this study provides a foundational tool to support evidence-informed policies, effective planning, and community-level interventions that contribute directly to the achievement of SDG 3 and to the broader strengthening of mental health care in Bolivia. Therefore, this study aimed to conduct the translation, cultural adaptation and content validity assessment of an instrument designed to comprehensively assess the construct of mental health literacy in urban Bolivian context, targeting two key populations: the general population and primary healthcare professionals.

## 2 Materials and methods

### 2.1 Study design

**Figure 1:**
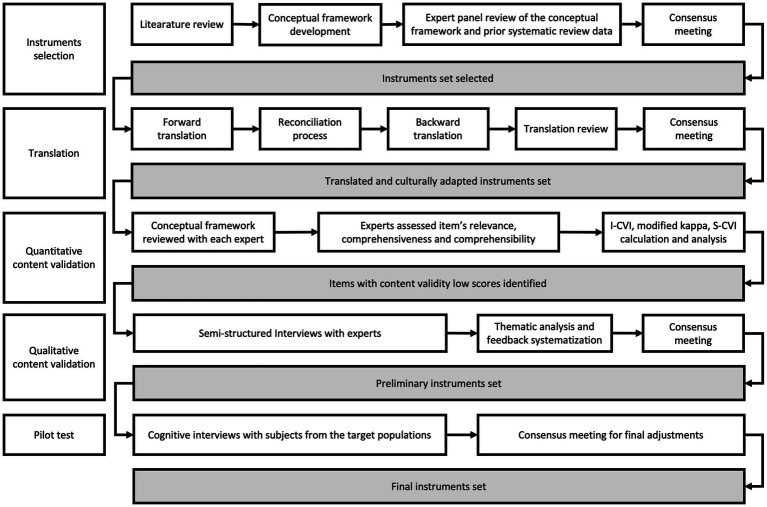
Flowchart illustrating the process of translation, cultural adaptation, and content validation of MHL instruments.

The study employed a mixed-methods design, integrating both quantitative and qualitative phases. Figure 1 shows the flowchart of the entire process.

### 2.2 Instruments selection

The process began with a comprehensive literature scoping review conducted in MEDLINE and APA PsycInfo, aimed to identify and synthesize instruments developed to assess MHL in the general population, describe their main characteristics, and analyze their psychometric properties. This review included studies published between 2010 and 2024 in English or Spanish, and used a search strategy combining the following terms: ((“Mental Health”[Mesh]) AND “Health Literacy”[Mesh] AND literacy* AND Measure*)). This review has been submitted for publication separately, and the PRISMA flow diagram can be found in Supplementary material 1.

Afterwards a comprehensive MHL conceptual framework was developed and presented to a consensus panel, conformed by two health researchers with wide experience in mental health research and instrument validation. Based on the conceptual framework review, the researchers recommended using three instruments, that collectively addressed the key dimensions of the framework:

- The Mental Health Literacy Scale (MHLS) (16), is a 35-item questionnaire designed to assess knowledge and attitudes related to mental health. It covers six domains: disorder recognition, information-seeking, knowledge of risk factors, self-treatment, professional help, and help-seeking attitudes. Items are scored on 4- or 5-point Likert scales, with several reversed items. Total scores range from 35 to 160. This instrument was selected because it includes different dimensions relevant to a comprehensive conceptual framework of MHL;
- The Perceived discrimination and devaluation scale (PDDS) (34), is a 12-item questionnaire that assesses perceived stigma—specifically devaluation and discrimination—toward individuals with mental illness. Items are rated on a four-point Likert scale (1 = strongly disagree to 4 = strongly agree), with total scores ranging from 12 to 36. This instrument was selected for its focus on stigma as a core dimension of MHL.
- The Mental health problems recognition module (MHPR) (35), is a six-item instrument designed to assess the ability to correctly identify two defining characteristics of six mental health problems—depression, psychosis, alcoholism, anxiety, personality disorder, and suicidal ideation—from a set of seven options. Each item is scored only if both correct characteristics are selected, yielding a total score ranging from 0 to 12. This instrument was chosen because of the ease with which its vignette-based format assesses the ability to recognize mental health problems.

### 2.3 Translation and cultural adaptation

The translation followed established best practices for the translation and cultural adaptation of patient-reported outcome measures (36, 37), aiming to ensure both linguistic accuracy and cultural relevance within the Bolivian context. This process included the phases of Forward Translation, Reconciliation, Back Translation, Back Translation Review, Harmonization, Cognitive Debriefing, and Review of Cognitive Debriefing. In this section, all steps are described except those related to Cognitive Debriefing, which are detailed later in Section 2.5.

The process began with forward translation of the two instruments originally developed in English, in which two independent bilingual translators—both native Spanish speakers—translated the original English instruments into Spanish. The translators were provided with background information on the MHL conceptual framework and were instructed to preserve the original meaning of the items while adapting them appropriately to the local context. A reconciliation phase followed, involving the two translators, a review panel (Experts panel 1) composed of the lead researcher, and two mental health research experts. These experts are medical professionals with postgraduate training in public health and approximately 20 years of experience in health research. The first expert, based outside of Bolivia, also holds postgraduate qualifications in mental health and a doctorate in medical sciences. This expert has led national and international research initiatives in mental health, including the development and validation of mental health literacy assessment instruments targeting general population outside of Bolivia. The second expert, a Bolivian national residing in Bolivia, holds postgraduate degrees in epidemiology and a doctorate in health research. This expert has participated in multiple mental health research projects in different settings and has contributed to the development and validation of mental health literacy assessment tools. This panel reviewed both versions to resolve discrepancies and agree on a single, harmonized translation.

The reconciled Spanish version then underwent backward translation by a bilingual translator—an English native speaker with no prior exposure to the original instruments—who translated the content conceptually back into English. This version was reviewed by the lead researcher and a mental health expert to confirm conceptual equivalence with the original version. To finalize the process, a consensus meeting was held with the Experts panel 1. The panel evaluated the forward and backward translations of the instruments originally developed in English, as well as the mental health problem recognition module originally written in Spanish, offering input on terminology, phrasing, and cultural relevance to ensure the instrument’s clarity and appropriateness for the Bolivian context.

### 2.4 Content validation

Content validation was performed in accordance with COSMIN (Consensus-based Standards for the selection of health Measurement Instruments) guidelines (38), focusing on three predefined domains: relevance, comprehensiveness, and comprehensibility of each item across the set of instruments. This process involved a two-phase approach: first, a quantitative survey, followed by in-depth qualitative interviews as described in the Content-validity assessment protocol available in Supplementary material 2. Both phases involved a panel of eight individuals identified as Experts panel 2, or “experts by experience”. The panel was composed of two representatives from each of the following areas of expertise: (a) specialized clinical mental health care; (b) research and validation of mental health assessment instruments; (c) primary care, including the management of mental health problems; (d) experiential expertise, defined as individuals with personal or family experience as patients affected by mental health problems. To qualify as an expert, participants were required to have a minimum of 5 years of experience in an aforementioned domain.

#### 2.4.1 Quantitative phase

In this phase, following the informed consent process, the principal investigator conducted individual sessions with each expert to review the conceptual framework related to MHL and the specific variables addressed by each item across the instruments. Particular emphasis was placed on key characteristics such as the target populations—namely, the general public and primary health care personnel in the urban Bolivian context—and the non-technical approach used to assess MHL, intended to establish a common baseline of understanding across both groups.

Subsequently, experts received the full set of instruments in the form of a digital questionnaire and were asked to rate each item across three predefined dimensions—relevance, comprehensiveness, and comprehensibility—using a 4-point Likert scale. For instance, the scale for relevance included: 1 = not relevant, 2 = somewhat relevant, 3 = quite relevant, and 4 = highly relevant. Following data collection, the Item-level Content Validity Index (I-CVI) was calculated for each item. The I-CVI represents the proportion of experts who rated an item as either 3 or 4, indicating the level of agreement regarding the COSMIN domains of relevance, comprehensiveness and comprehensibility. For a number of 8 experts, as is the case in this study, I-CVI values ≥ 0.78 are considered to show acceptable agreement between experts (39).

Following established methodological recommendations with specific calculation examples (39, 40), a modified kappa index was calculated for each item to adjust the I-CVI values for chance agreement and to better assess expert consensus across the three evaluated dimensions in relation to the target construct. The computation of the modified kappa requires estimating the probability of a chance occurrence, which is derived using the binomial random variable formula, with one specific outcome:


$$\mathrm{pc}=[N!/A!(N-A)!]*0.5^{N}$$

Where: N = number of experts, and A = Number of experts agreeing on good relevance (3 and 4 scores).

Afterwards, the modified kappa index (k*) is calculated using the formula:


$$k^{*}=(\text{ICVI}-\mathrm{pc})/(1-\mathrm{pc})$$

The results for k* were interpreted using the thresholds proposed by Cicchetti and Sparrow (41) and Fleiss (42): poor (<0.4), fair (0.4–0.59), good (0.6–0.74), and excellent (≥0.75). Items that received scores below “optimal values” for I-CVI (≥ 0.78) and k* (≥0.75) were flagged as indicating low interrater consistency in assessing the three dimensions defined by COSMIN. These items were subsequently identified for further review during the qualitative content validity phase of the study, where final decisions were made regarding whether they would be revised or discarded.

Additionally, Scale-level Content Validity Indexes (S-CVI) were calculated. These included the Universal Agreement among experts (S-CVI/UA), defined as the proportion of items rated as 3 or 4 by all experts for a given construct or the entire instrument, reflecting complete consensus among evaluators. A value of 0.80 or higher is recommended as indicative of acceptable content validity (43, 44). To illustrate the calculation of the S-CVI/UA, Table 1 presents the relevance ratings provided by eight experts for a 12-scale. Following one of the different calculation methods proposed by Polit (43), the number of items with universal agreement (i.e., items rated as 3 or 4 by all experts) is divided by the total number of items in the scale. This calculation method is used in this document.

**Table 1:** Relevance ratings and expert agreement for the calculation of Content Validity Indexes.

Item	Experts’ ratings								Experts in agreement	I-CVI
	E1	E2	E3	E4	E5	E6	E7	E8		
1	X	–	X	X	–	X	X	X	6	0.75
2	X	X	X	X	X	X	X	X	8	1.00
3	–	X	X	X	–	X	X	X	6	0.75
4	–	X	X	X	X	–	X	X	6	0.75
5	X	X	X	X	X	X	X	X	8	1.00
6	X	X	X	X	X	X	X	X	8	1.00
7	X	X	X	X	X	–	X	X	7	0.88
8	X	X	X	X	X	–	X	X	7	0.88
9	X	X	X	X	–	X	X	X	7	0.88
10	X	X	X	X	X	–	X	X	7	0.88
11	X	X	X	X	X	X	X	X	8	1.00
12	X	X	X	X	X	X	X	X	8	1.00

Item-level Content Validity Index (I-CVI); (X) indicates that the was rated 3 or 4 by an expert on a 4-point Likert scale (1 = not relevant, 4 = highly relevant); (−) indicates that the was rated 1 or 2 by an expert on the same 4-point Likert scale.

Using the data presented in Table 1, only 5 of the 12 items received relevance ratings of 3 or 4 from all eight experts. Consistent with the definition requiring universally congruent ratings across experts, the resulting S-CVI/UA in this example is 0.42, as shown in the following formula:


$$\begin{array}{l} S-\mathrm{CVI}/\mathrm{UA}=\text{Items with universal agreement}/\\ \text{Total number of items} \end{array}$$

Where:


S−CVI/UA=5/12

S−CVI/UA=0.42

On the other hand, the Average content validity index (S-CVI/Ave) was also calculated. This index represents the mean of the I-CVI across all items in the scale for a specific construct or the entire instrument. Values equal to or greater than 0.90 interpreted as indicative of excellent content validity (43, 44). To illustrate the calculation of the S-CVI/Ave, using the I-CVI data presented in Table 1 and following the calculation method proposed by Polit (43), the I-CVI values for all items in the scale are summed and then divided by the total number of items, obtaining a result of 0. 90 as shown in the following formula.


S−CVI/Ave=SumofallI−CVIvalues/Total number of items

Where:


$$S-\mathrm{CVI}/\mathrm{Ave}=(\begin{array}{l} 0.75+1+0.75+0.75+1+1+0.88+\\ 0.88+0.88+0.88+1+1 \end{array})/12$$

S−CVI/Ave=0.90

Finally, to complement the analysis of the instrument’s quality, the Fernández-Huerta (FH) index was applied as a measure of readability for the entire instrument. The FH index is a Spanish-language adaptation of the Flesch Reading Ease formula and evaluates textual readability by analyzing the relationship between sentence length and syllable density in a 100-word segment (45). The formula is expressed as follows:


FH=206.84−0.60t−1.02s

Where t represents the number of syllables and s the number of sentences in a 100-word sample. The resulting score is interpreted using the following intervals: 0–30 = Very difficult, 30–50 = Hard, 50–60 = Somewhat hard, 60–70 = Normal, 70–80 = Somewhat easy, 80–90 = Easy, and 90–100 = Very easy (46). Higher scores indicate greater readability.

#### 2.4.2 Qualitative phase

In the second phase of content validity assessment, semi-structured interviews were conducted via teleconference with the same panel of eight experts, following established guidelines to assess the content validity of existing instruments (47), and using an interview guide based on the three measurement properties suggested by COSMIN, available in Supplementary material 3. The interviews were conducted (47) by the principal investigator, who has training and experience in qualitative research. Each interview lasted about 1 h, was recorded, transcribed verbatim, and analyzed thematically to identify key themes and recurrent patterns. The aim was to refine items with lower ratings in relevance, comprehensiveness, and comprehensibility, and to improve the overall instrument in accordance with COSMIN guidelines (38). During this phase, experts were asked to evaluate whether each item aligned with the three dimensions recommended by COSMIN—relevance, comprehensiveness, and comprehensibility—and, based on the definitions of these dimensions, to determine whether the item should be retained, modified, or removed. Two members of Expert Panel 1, both with extensive experience in qualitative research, conducted the data analysis. They drew on the theoretical framework of the three COSMIN measurement properties and their characteristics to guide the coding approach, using triangulation as the primary strategy to ensure the quality and rigor of the analytic process (48). Insights from these interviews informed revisions made by the principal investigator and consensus panel to ensure content validity and cultural adaptation.

### 2.5 Pilot testing

As the final phase of the translation and cultural adaptation process recommended by methodological guidelines (36, 37), the pilot testing of the instrument, following quantitative and qualitative content validation, was conducted through a cognitive debriefing process with participants from both target groups, aiming to “highlight any items that may be inappropriate at a conceptual level” as well as to identify possible confusion sources. Thus, the revised instrument, was pilot-tested with a sample of 10 individuals—five health professionals and five members of the general population from an urban setting. This pilot aimed to assess the instrument’s comprehensiveness, comprehensibility and usability in real-world settings. Cognitive interviews were conducted to gather participants’ feedback on their understanding of the items, ease of completion, and overall clarity of the instrument, using the think-aloud and verbal probing techniques (49).

## 3 Results

### 3.1 Instrument selection

The conceptual framework for MHL proposed that, beyond traditional dimensions—such as knowledge of mental health issues and attitudes toward help-seeking—assessment should also include dimensions such as stigma evaluation and recognition of mental health symptoms. Moreover, the framework emphasized the need for mental health assessment not only in the general population but also among healthcare personnel, particularly those in primary care settings. This would enable effective communication between these groups and help strengthen the knowledge, attitudes, and practices surrounding mental health care. This rationale, combined with insights from the previous systematic review and expert recommendations, guided the selection of the instruments used in this study.

### 3.2 Translation

The translation process resulted in a preliminary Spanish version that preserved a high level of semantic and conceptual equivalence with the original English version. Following the stages of forward translation, reconciliation, back translation, translation review, and consensus meeting, most items were translated without significant changes to their meaning.

### 3.3 Quantitative content validation

The expert panel 2 involved in this process consisted of eight participants: One psychiatrist and one psychologist in the group specialized in clinical mental health care; one psychiatrist and one psychologist in the group with experience in research and validation of mental health assessment instruments; one physician and one psychologist in the primary care group; and one teacher and one domestic worker in the experiential expertise group, resulting in a majority of experts being psychologists (37.5%), followed by psychiatrists (25.0%). Of the panel, 62.5% were women, with ages ranging from 32 to 68 years (Mean = 45.63, SD = 9.52). Most participants (87.5%) held at least a bachelor’s degree. All had over 5 years of experience in either mental health research, clinical work, or lived experience with a diagnosed mental health problem. The following information was derived from the contributions of the expert panel.

#### 3.3.1 Item-level content validity index

Table 2 shows the distribution of items in the three evaluation categories proposed by Cicchetti and Sparrow (41) based on the scores obtained in the calculation of the modified kappa index (k*). Information is shown for each of the instruments evaluated in the three dimensions evaluated (relevance, comprehensiveness, and comprehensibility), detailed results for each instrument can be found in the Supplementary Files S1–S3.

**Table 2:** Item classification by modified kappa index across instruments and dimensions.

Instrument	Items evaluation	Relevance		Comprehensiveness		Comprehensibility	
		*n*	%	*n*	%	*n*	%
MHLS	Excellent	23	65.71	17	48.57	22	62.86
	Good	11	31.43	9	25.71	10	28.57
	Fair	1	2.86	9	25.71	3	8.57
PDDS	Excellent	9	75.00	10	83.33	9	75.00
	Good	3	25.00	2	16.67	3	25.00
	Fair	0	0.00	0	0.00	0	0.00
MHPR	Excellent	6	100.00	6	100.00	6	100.00
	Good	0	0.00	0	0.00	0	0.00
	Fair	0	0.00	0	0.00	0	0.00
All three instruments	Excellent	38	71.70	33	62.26	37	69.81
	Good	14	26.42	11	20.75	13	24.53
	Fair	1	1.89	9	16.98	3	5.66

MHLS, Mental health literacy scale; PDDS, Perceived discrimination and devaluation scale; MHPR, Mental health problems recognition.

The relevance dimension shows strong content validity across instruments, with MHPR achieving 100% excellent ratings. PDDS also performed well, with 75% of items rated excellent and no fair ratings. MHLS had 65.7% excellent and 2.9% fair items. Overall, 97.14% of all items were rated good or excellent, indicating that the instruments are highly relevant for assessing mental health literacy in the target context.

In the comprehensiveness dimension, MHPR achieved 100% excellent ratings. PDDS also showed strong performance with 83.33% excellent and no fair ratings. MHLS had a more varied distribution, with 48.57% excellent but 25.71% fair ratings, suggesting areas for improvement. Overall, 83.02% of all items were rated with good or excellent scores, indicating that the items generally cover the construct in a comprehensive manner.

For the comprehensibility dimension, MHPR achieved a perfect score with 100% excellent ratings. PDDS also showed strong results, with 75% excellent and 25% good ratings. MHLS demonstrated solid performance, with 62.86% excellent and 8.57% fair ratings. Overall, 94.34% of all items were rated good or excellent, indicating that the items were considered comprehensible for the target populations.

It is noteworthy that none of the items were rated as poor across any of the three dimensions, and only 13 items received a rating of fair. To enhance the validity of the instrument, all items rated as fair or good—a total of 24—were flagged for more in-depth analysis in the subsequent phase of the study.

#### 3.3.2 Scale-level content validity index

Regarding the scale-level content validity indices, the complete set of 53 items obtained an S-CVI/UA of 0.32 for relevance, 0.25 for comprehensiveness, and 0.32 for comprehensibility. These values fall well below the recommended threshold of 0.80, indicating a lack of universal agreement among experts and suggesting considerable variability in their evaluations across all three domains. In contrast, the S-CVI/Ave for all the items were 0.88 for relevance, 0.84 for comprehensiveness, and 0.87 for comprehensibility. Although these values do not reach the optimal criterion of 0.90 for excellent content validity, they indicate moderate consensus among experts regarding the quality of the items. These findings reflect general agreement but also highlight the need for targeted refinement of specific items.

Each set of items derived from the source instruments was also evaluated individually using the scale-level indices. For the items adapted from the MHLS, the S-CVI/UA was 0.34 for relevance, 0.23 for comprehensiveness, and 0.34 for comprehensibility, again indicating low levels of universal agreement. The corresponding S-CVI/Ave values were 0.87, 0.81, and 0.86, respectively, suggesting an acceptable level of agreement but falling short of the ideal threshold for excellent content validity. These results point to a need for item revision, particularly to enhance content coverage and clarity.

The items derived from the PDDS showed higher S-CVI/UA values of 0.42 across all three domains, which, while still below the 0.80 criterion, represent relatively stronger consensus compared to the other instruments. Notably, the S-CVI/Ave values for the PDDS items were 0.90 for relevance, 0.91 for comprehensiveness, and 0.90 for comprehensibility—meeting the threshold for excellent content validity in all three domains. This suggests that the PDDS items were well-aligned with the constructs assessed and well-understood by the expert panel.

Conversely, the items based on the MHPR obtained an S-CVI/UA of 0.00 across all domains, reflecting a complete lack of universal agreement among experts regarding the relevance, comprehensiveness, and comprehensibility of these items. Despite this, the S-CVI/Ave values for these items were 0.88 in all three domains, indicating acceptable but not excellent levels of agreement. These mixed results suggest that while there was general expert consensus on the adequacy of these items, the lack of full agreement points to potential ambiguities or content gaps that should be addressed. Detailed information on the S-CVI/UA and S-CVI/Ave values obtained for each construct can be found in the Supplementary material S4–S6.

#### 3.3.3 Readability metrics

The FH readability score for the complete instrument was 70.87, which corresponds to a classification of “somewhat easy” according to established interpretation thresholds and linked to a 7th grade educational level (50). This suggests that the instrument is generally accessible to a broad audience, requiring only a moderate level of reading proficiency for adequate comprehension. Additional readability metrics further support this assessment: the instrument exhibited an average of 4.95 letters per word and 2.1 syllables per word, indicating a moderate level of lexical complexity. Sentence structure was also relatively simple, with an average of 9.77 words per sentence, suggesting that syntactic complexity is low. Moreover, only 13.52% of the words were polysyllabic (i.e., containing four or more syllables), a proportion that aligns with texts intended for general populations.

### 3.4 Qualitative content validation

First, in this phase experts provided feedback on the 24 items that received “not optimal” ratings in the quantitative phase, offering suggestions for improvement. Second, their perceptions of the overall structure and dimensions of the instrument were explored, along with recommendations to enhance their clarity and relevance.

In relation to the items that received “non-optimal” ratings in the quantitative phase, an important finding was that experts suggested the low scores could be attributed to the use of specific mental health terminology (e.g., dysthymia, agoraphobia), which could potentially difficult these terms understanding for general population in the local context. However, they also emphasized that, considering the purpose of the instruments, these items remained relevant and comprehensive for assessing MHL different dimensions. Feedback and specific suggestions related to all 24 low-scoring items are presented in Table 3.

**Table 3:** Low-rated items and expert recommendations for revision.

Items original wording	Initial translation wording	Experts’ feedback, specific suggestions and modification rationale	Items modified wording
4. To what extent do you think it is likely that Personality Disorders are a category of mental illness. 5. To what extent do you think it is likely that Dysthymia is a disorder 6. To what extent do you think it is likely that the diagnosis of Agoraphobia includes anxiety about situations where escape may be difficult or embarrassing. 9. To what extent do you think it is likely that in general in Australia, women are MORE likely to experience a mental illness of any kind compared to men. 10. To what extent do you think it is likely that in general in Australia, men are MORE likely to experience an anxiety disorder compared to women 13. To what extent do you think it is likely that Cognitive Behavior Therapy (CBT) is a therapy based on challenging negative thoughts and increasing helpful behaviors.	4. ¿En qué medida cree que es probable que los trastornos de la personalidad sean un tipo de enfermedad mental? 5. ¿En qué medida cree que es probable que la Distimia sea un trastorno? 6. ¿En qué medida cree que es probable que el diagnóstico de agorafobia incluya ansiedad ante situaciones en las que escapar puede ser difícil o vergonzoso? 9. ¿En qué medida cree que es probable que, en general en Bolivia, las mujeres tengan MAS probabilidades de experimentar una enfermedad mental de cualquier tipo en comparación con los hombres? 10. ¿En qué medida cree que es probable que, en general en Bolivia, los hombres tengan MAS probabilidades de experimentar un trastorno de ansiedad en comparación con las mujeres? 13. ¿En qué medida cree que es probable que la terapia cognitivo-conductual (TCC) sea una terapia basada en desafiar los pensamientos negativos y aumentar las conductas útiles?	For these items, as well as others within the same instrument, it was recommended to modify the initial phrasing to a more natural and contextually appropriate Spanish equivalent of “How likely is it that.” Additionally, it was observed that the use of specific mental health terminology in this and related items may have contributed to the low scores obtained during testing. Nevertheless, the phrasing was deemed appropriate for assessing the baseline knowledge of the target populations regarding mental health concepts.	4. ¿Qué tan probable es que los Trastornos de personalidad sean un tipo de enfermedad mental? 5. ¿Qué tan probable es que la Distimia sea un problema de salud mental? 6. ¿Qué tan probable es que el diagnóstico de Agorafobia incluya ansiedad ante situaciones en las que escapar puede ser difícil o vergonzoso? 9. ¿Qué tan probable es que, en Bolivia las mujeres tengan MÁS probabilidades de experimentar una enfermedad mental de cualquier tipo, en comparación con los hombres? 10. ¿Qué tan probable es que, en Bolivia los hombres tengan MÁS probabilidades de experimentar un trastorno de ansiedad en comparación con las mujeres? 13. ¿Qué tan probable es que la Terapia Cognitivo Conductual (TCC) sea una terapia basada en desafiar los pensamientos negativos y aumentar las conductas útiles?
11. To what extent do you think it would be helpful for someone to improve their quality of sleep if they were having difficulties managing their emotions (e.g., becoming very anxious or depressed). 12. To what extent do you think it would be helpful for someone to avoid all activities or situations that made them feel anxious if they were having difficulties managing their emotions.	11. ¿En qué medida cree que sería útil para alguien mejorar su calidad de sueño si tuviera dificultades para gestionar sus emociones (por ejemplo, si está muy ansioso o deprimido)? 12. ¿En qué medida cree que sería útil para alguien evitar todas las actividades o situaciones que le hicieran sentir ansioso, si tuviera dificultades para gestionar sus emociones?	For these items, it was recommended to modify the initial phrasing to a more natural and contextually appropriate Spanish equivalent of “How useful would it be.”.	11. ¿Qué tan útil sería para alguien que tuviera dificultades para manejar sus emociones, mejorar su calidad de sueño (por ejemplo, si está muy ansioso o deprimido)? 12. ¿Qué tan útil sería para alguien que tuviera dificultades para manejar sus emociones, evitar todas las actividades o situaciones que le hicieran sentir ansioso?
14. To what extent do you think it is likely that the following is a condition that would allow a mental health professional to break confidentiality: If you are at immediate risk of harm to yourself or others 15. To what extent do you think it is likely that the following is a condition that would allow a mental health professional to break confidentiality: If your problem is not life-threatening and they want to assist others to better support you	14. ¿En qué medida es probable que lo mencionado a continuación sea una condición que permitiría a un profesional de la salud mental romper la confidencialidad? Si usted corre riesgo inmediato de hacerse daño a sí mismo o a otras personas. 15. ¿En qué medida es probable que lo mencionado a continuación sea una condición que permitiría a un profesional de la salud mental romper la confidencialidad? Si su problema no pone en peligro la vida, y quieren ayudar a otros a brindarle un mejor apoyo.	For these items, it was noted that the Spanish wording was ambiguous and lacked sufficient contextual cues, making it unclear that it is the health professional who is responsible for assessing the risk. Additional clarification was recommended to ensure accurate interpretation by respondents.	14 ¿Qué tan probable es, que a un profesional de la salud mental se le permita romper la confidencialidad en la siguiente situación?: Si el profesional considera que usted (como paciente) corre riesgo inmediato de hacerse daño a sí mismo o a otras personas 15. ¿Qué tan probable es, que a un profesional de la salud mental se le permita romper la confidencialidad en la siguiente situación?: Si el profesional considera que usted (como paciente) tiene un problema que no pone en peligro su vida, pero quiere avisarles a otras personas de ese problema para que ellos le brinden un mejor apoyo
19. I am confident I have access to resources (e.g., GP, internet, friends) that I can use to seek information about mental illness.	19. Estoy seguro de tener acceso a recursos (por ejemplo, médico general, Internet, amigos) que puedo utilizar para buscar información sobre enfermedades mentales.	For this item, as well as others within the same instrument, the phrase “I am confident” was found to be somewhat ambiguous in Spanish due to the existence of multiple possible translations. It was therefore recommended to consider using the word “confianza (trust)” to more accurately convey the intended meaning of the original expression.	19. Me siento confiado de disponer de personas y recursos (p. ej., médico general, Internet, amigos) con los que puedo buscar información sobre enfermedades mentales.
20. People with a mental illness could snap out if it if they wanted. 21. A mental illness is a sign of personal weakness. 22. A mental illness is not a real medical illness. 23. People with a mental illness are dangerous. 24. It is best to avoid people with a mental illness so that you do not develop this problem. 26. Seeing a mental health professional means you are not strong enough to manage your own difficulties. 27. If I had a mental illness, I would not seek help from a mental health professional. 30. How willing would you be to spend an evening socializing with someone with a mental illness? 34. How willing would you be to vote for a politician if you knew they had suffered a mental illness?	20. Las personas con una enfermedad mental podrían recuperarse si quisieran. 21. Una enfermedad mental es un signo de debilidad personal. 22. Una enfermedad mental no es una enfermedad médica real. 23. Las personas con una enfermedad mental son peligrosas. 24. Es mejor evitar a las personas con una enfermedad mental para no desarrollar este problema. 26. Ver a un profesional de la salud mental significa que no tienes la fuerza suficiente para gestionar tus propias dificultades. 27. Si tuviera una enfermedad mental, no buscaría ayuda de un profesional de la salud mental. 30. ¿Qué tan dispuesto estaría a pasar una noche socializando con alguien que tiene una enfermedad mental? 34. ¿Qué tan dispuesto estaría a votar por un político si supieras que ha sufrido una enfermedad mental?	For these items, it was noted that the use of absolute statements might be perceived by respondents as too direct when addressing potentially taboo subjects. However, there was consensus that, when intended to assess the baseline level of discriminatory attitudes, the questions are appropriate and acceptable for use with the target populations. In this regard, the items related to this characteristic did not require modification and were retained in the original wording of the translated version.	20. Las personas con una enfermedad mental podrían recuperarse si quisieran. 21. Una enfermedad mental es un signo de debilidad personal. 22. Una enfermedad mental no es una enfermedad médica real. 23. Las personas con una enfermedad mental son peligrosas. 24. Es mejor evitar a las personas con una enfermedad mental para no desarrollar este problema. 26. Ver a un profesional de la salud mental significa que no eres lo bastante fuerte para manejar tus propias dificultades. 27. Si tuviera una enfermedad mental, no buscaría ayuda de un profesional de la salud mental. 30. ¿Qué tan dispuesto estaría a pasar una noche socializando con alguien que tiene una enfermedad mental? 34. ¿Qué tan dispuesto estaría a votar por un político si supiera que ha sufrido una enfermedad mental?
36. Most people would willingly accept a former mental patient as a close friend 37. Most people believe that a former mental patient is just as trustworthy as the average citizen 39. Most people would accept a fully recovered former mental patient as a teacher of young children in a public school	36. La mayoría de las personas estaría dispuesta a aceptar de buena gana a un ex paciente mental como amigo cercano. 37. La mayoría de las personas cree que un ex paciente mental es tan confiable como el ciudadano promedio. 39. La mayoría de las personas aceptaría a un ex paciente mental completamente recuperado como maestro de niños pequeños en una escuela pública.	For this group of questions, it was noted that the Spanish translation of the term “former mental patient” might lack clarity for respondents. Therefore, it was suggested to replace it with the more explicit phrasing “a person who had a mental health problem”.	36. La mayoría de la gente aceptaría de buena gana a una persona que tuvo un problema de salud mental como amigo cercano. 37. La mayoría de la gente cree que una persona que ha estado en un hospital psiquiátrico es tan inteligente como la persona promedio. 39. La mayoría de la gente aceptaría a una persona que tuvo un problema de salud completamente recuperada, como maestro de niños pequeños en una escuela pública.
42. Most people think less of a person who has been in a mental hospital	42. La mayoría de las personas piensa menos de una persona que ha estado en un hospital psiquiátrico.	For this item, the Spanish translation of the phrase “think less” was considered potentially ambiguous and confusing. It was therefore recommended to use the word “menosprecia (Looks down on),” as it more accurately conveys the intended meaning of holding someone in lower regard.	42. La mayoría de la gente menosprecia a una persona que ha estado en un hospital psiquiátrico.

Below are representative excerpts from the experts’ interviews [translated from Spanish], highlighting their conclusions and recommendations regarding the relevance, comprehensibility, and understanding of the instrument and its items.

Regarding the overall instrument relevance, experts agreed that the items appropriately reflected the construct of interest—mental health literacy (MHL)—and were suitable for assessing baseline MHL levels in both target populations: the general public and primary healthcare professionals. They also considered the items to be contextually appropriate for the settings in which the instruments are intended to be applied.

*“(Commenting about the instruments’ relevance for the context) It's good because today… from a current perspective, I can say that you are indeed addressing all the situations that are occurring today in Bolivia… and in South America” (Exp. 3).*

*“(Commenting about the items’ recall period) The memories came back to me like this (quick hand gesture), almost in a rush... I immediately had the question and the example, so the evocation of the moment of the answer is appropriate.” (Exp. 8).*

In terms of comprehensiveness, experts reported that the set of instruments adequately covered all relevant dimensions and concepts, with no major omissions that would limit its capacity to assess the construct.

*"(Commenting on possible missing items or dimensions) I don’t think anything is missing. From what I understand about mental health literacy, everything that was intended to be included is being reviewed." (Exp. 6).*

Finally, concerning comprehensibility, the experts indicated that the response options were appropriate for the items and that, following minor adjustments, the instructions were clear and understandable for the intended populations.

*"(Commenting about items’ comprehensibility) Indeed, even if some people don't recognize certain terms, the way the questions are asked and the answer options are understandable to the target populations." (Exp. 2).*

After analyzing and systematizing the observations and suggestions related to individual items and the questionnaire as a whole, the conciliation panel and the principal investigator reviewed them and made the necessary adjustments to the identified items and the wording of the response options. The changes made based on the experts’ suggestions resulted in a significantly improved instrument in terms of item clarity, response option suitability, and overall structural coherence. Overall, no items were recommended for elimination; instead, minor wording modifications were made to reduce ambiguity and improve clarity and ease of interpretation.

### 3.5 Pilot testing

At this stage, the instrument—previously refined through the incorporation of expert recommendations and suggestions during the qualitative phase—was subjected to a pilot test. The pilot sample consisted of 10 participants, 50% of whom were women. The age range was 25–56 years (*M* = 41.5, SD = 10.45). The health personnel group included three physicians and two nurses, while the general population group comprised two individuals with professional degrees and three with only completed secondary education.

Participants did not report significant concerns regarding the completeness, relevance, or comprehensibility of the items. Most respondents found the questions to be clear and easy to understand, with no major issues raised during the administration process. Following the pilot test, the results were reviewed by the Experts panel 1. Based on this review, it was concluded that the instrument performed adequately in the target population. Minor lexical and syntactic adjustments were made to 10 items to enhance clarity and comprehensibility, without altering the underlying constructs. No substantial changes to the content or structure of the instrument were deemed necessary.

The final Spanish version consists of 53 items that are conceptually equivalent to the original instruments, with wording that balances technical accuracy and clarity for the general public. Eighteen items out of the total have reverse scoring in accordance with their original formulation; these items are duly identified in the final instrument, available as Supplementary material 7. While preserving the overall format of the original instruments, the structure of the instrument’s constructs is briefly described below:

- Recognition of Disorders (8 items measured on a 4-point Likert scale),
- Knowledge of Risk factors and causes (2 items measured on a 4-point Likert scale)
- Self-Treatment Knowledge (2 items measured on a 4-point Likert scale)
- Knowledge of Professional help available (3 items measured on a 4-point Likert scale)
- Knowledge of How to seek mental health information (4 items measured on a 5-point Likert-scale).
- Attitudes that Promote recognition and appropriate help-seeking (16 items measured on a 5-point Likert scale)
- Self-reported perceived discrimination and devaluation towards mental illness (12 items measured on a 4-point Likert scale).
- Recognition of Disorders symptoms (6 items assessing the identification of 2 correct symptoms from a set of seven options).

### 3.6 Ethical considerations

The project was reviewed and approved by two research ethics committees: the Research Ethics Committee of the National Institute of Public Health (INSP) in Mexico, and the Research Ethics Committee of the Medicine School at the Universidad Mayor de San Simón in Cochabamba, Bolivia. The study was conducted in accordance with international recommendations for health research.

The consent scripts were reviewed and approved by the ethics committees, taking into account their comprehension by individuals with low literacy. Since the recruitment of experts was conducted remotely, it was not possible to obtain signed consent in the presence of others. Therefore, Zoom meetings were used to obtain verbal consent. The recordings of the informed consent process and the qualitative interviews were coded and securely stored on a password-protected hard drive with restricted access.

## 4 Discussion

This study applied a rigorous content validation assessment process using not only the traditional relevance dimension, but also comprehensiveness, and comprehensibility following COSMIN criteria (51). Framed within a robust conceptual model of MHL, eight experts with diverse professional and experiential backgrounds participated, all from an urban setting, ensured varied perspectives for the process. This multidimensional approach guided both the selection and content validation assessment of instruments to assess MHL in the general population and primary care personnel from urban context—two key groups that must share a common language to strengthen mental health care delivery as previous experiences have explored (52), especially in contexts with a strong need for mental health care improvement as Bolivia.

This study not only contributes a content-validated tool for assessing MHL in the Bolivian context, but also advances regional capacity for instrument adaptation in under-researched Latin American settings. Unlike prior efforts focused on general translation or partial content validity assessment, this work integrates conceptual, linguistic, and cultural adaptation through a rigorous, multidimensional process. This approach can serve as a model for similar efforts in other low- and middle-income countries (LMICs), particularly those with multiethnic populations and limited mental health infrastructure.

In the quantitative content validity phase, the combination of the I-CVI and the modified kappa statistic was considered the most appropriate to assess the instruments content validity. This method is especially appropriate when involving a small panel of experts. Moreover, it quantifies the level of agreement for each item while accounting for chance agreement, and its results are easily interpretable, considering scores below “optimal values” for I-CVI (≥ 0.78) and k* (≥0.75). These characteristics enhance the methodological rigor of the analysis for this study, in comparison to other approaches as Lawshes CVR or other options, as commented by different authors (39, 44, 53). The results showed strong content validity across most items at the I-CVI assessment. No item was rated as poor in any of the three assessed dimensions. Items coming from the MHPR and PDDS showed particularly high ratings across all dimensions. Items from the MHLS showed more variability, especially in comprehensiveness, with 25.71% of items rated as fair, often due to the use of complex terminology or subtle linguistic nuances. Nonetheless, these issues were largely addressed in the qualitative phase through targeted revisions.

In relation to the S-CVI analysis, the results indicate that, although the average agreement among experts was generally acceptable across most domains and instruments, S-CVI/UA agreement was limited and fell below thresholds. Regarding this limitation, it is important to note that as the number of evaluators increases, it becomes increasingly difficult to achieve positive results in terms of universal agreement (39, 43). These findings underscore areas within the instrument that would benefit from further refinement to enhance clarity, content coverage, and alignment with the intended constructs.

The subsequent qualitative phase of content validation played a key role in this process, as it allowed for an in-depth examination of the areas for improvement identified through the analysis of the I-CVI results during the quantitative phase. This qualitative phase informed the decisions regarding the revision, retention, or elimination of specific items in the instrument.

Regarding the readability metrics, the indicators measured demonstrate that the instrument’s language is appropriately tailored to the intended audience, favoring clarity and ease of understanding—key elements for ensuring accurate responses and reducing respondent burden in survey-based research.

The qualitative phase strengthened the content validity of the instrument in accordance with previous recommendations (54). This phase began by exploring the underlying reasons behind the lower quantitative ratings. Key feedback centered on terminology, phrasing clarity, and cultural appropriateness, recommending a more natural phrasing in Spanish and the inclusion of contextual cues. Despite some challenges, all 24 flagged items were retained due to their conceptual relevance, with refinements made to enhance clarity and acceptability. Secondly, the experts’ assessments regarding the overall relevance, comprehensiveness, and comprehensibility of the instrument and its dimensions—guided by the COSMIN criteria—reflected a strong consensus on its conceptual adequacy.

Following synthesis of both phases, no items were removed, maintaining the conceptual coverage of the original instruments. This iterative process ensured linguistic precision and cultural adaptation, allowing the tool to reliably measure baseline MHL. Importantly, the instrument is designed to assess populations who may not yet be familiar with mental health terminology as well as healthcare professionals. Thus, its current form is suitable for baseline assessments in both populations. However, for post-intervention evaluations, it will be essential to provide clear definitions of key terms to ensure that responses reflect improved understanding, rather than confusion due to unfamiliar language. This distinction is critical for valid measurement of MHL change over time.

### 4.1 Performance of MHL constructs in relation to content validity metrics

This section presents a construct-level analysis of content validity across the three evaluated dimensions—relevance, comprehensiveness, and comprehensibility—using item-level and scale-level metrics. By jointly considering I-CVI values, modified kappa coefficients, and S-CVI indicators, this analysis identifies both areas of strong expert consensus and domains that posed greater challenges during the validation process. Emphasis is placed on comparing the performance of individual constructs across source instruments, as well as on examining the concentration of items with suboptimal ratings, in order to inform targeted refinements and contextual alignment of the adapted instrument.

In the evaluation of the relevance dimension, the results indicate an overall favorable performance, although with variability across constructs. Constructs derived from the PDDS (Self-reported perceived discrimination and devaluation towards mental illness) and the MHPR (Recognition of disorders symptoms) showed the strongest performance, with all items achieving I-CVI values ≥ 0.78 and modified kappa coefficients predominantly in the excellent range. At the construct level, both reached S-CVI/Ave values close to or equal to 0.90, indicating a high degree of expert consensus regarding their conceptual relevance. In contrast, several constructs from the MHLS posed greater challenges, particularly Recognition of disorders and Attitudes that promote recognition and appropriate help-seeking, which concentrated a higher number of items with I-CVI values of 0.75 or lower and at least one item with a fair k* rating. Moreover, none of the MHLS constructs reached acceptable S-CVI/UA values (≥ 0.80), reflecting limited universal agreement among experts. These findings suggest that, although the content was generally perceived as conceptually relevant, certain MHLS domains required refinement to improve alignment with the target context.

The assessment of comprehensiveness revealed greater challenges compared with relevance, particularly within MHLS constructs. Domains such as Self-treatment knowledge, Knowledge of professional help available, and Attitudes that promote recognition and appropriate help-seeking included multiple items with I-CVI values below the acceptable threshold (≤ 0.75), several of which were associated with fair k* coefficients, suggesting perceived inadequacies in content coverage. At the scale level, these constructs exhibited low S-CVI/UA values (≤ 0.31) and S-CVI/Ave values below 0.90, indicating limited consensus regarding the sufficiency of their content. In contrast, the construct Knowledge of how to seek mental health information demonstrated the strongest performance in this dimension, with all items achieving optimal I-CVI and k* values, as well as an S-CVI/Ave close to 0.97. Similarly, the PDDS construct maintained high S-CVI/Ave values (≥ 0.91), reflecting consistent perceptions of adequate construct coverage. Overall, these results suggest that the most substantial comprehensiveness challenges were concentrated in broader or more abstract MHLS constructs.

In the comprehensibility dimension, the findings indicate generally acceptable performance, although notable differences across constructs were again observed. The strongest results were found for Knowledge of how to seek mental health information and for the PDDS and MHPR constructs, whose items consistently achieved I-CVI values ≥ 0.78 and k* coefficients in the good to excellent range. These constructs also showed high S-CVI/Ave values (approximately 0.90), reflecting strong expert agreement regarding clarity and ease of understanding. Conversely, several MHLS constructs presented greater difficulties, particularly Recognition of disorders and Knowledge of professional help available, where multiple items received I-CVI values between 0.63 and 0.75 and k* coefficients in the fair range. None of the MHLS constructs achieved acceptable S-CVI/UA values, suggesting a lack of universal agreement on the comprehensibility of all items within these domains. These findings indicate that the main limitations in this dimension were associated with the use of technical terminology and formulations that required linguistic adjustment to better fit the local context.

### 4.2 Strengths and limitations

A key strength of this study is its mixed-methods design, which combined quantitative and qualitative approaches to ensure rigorous content validation. Adherence to COSMIN guidelines enhanced methodological quality, while the inclusion of a diverse expert panel—comprising researchers, clinicians, primary care professionals, and individuals with lived experience—strengthened the contextual relevance of the instruments. The systematic translation and adaptation process was also a notable strength, as it implemented the best practice recommendations outlined by methodological guidelines (36, 37). Forward and backward translation, expert consensus, and cognitive interviews helped ensure both linguistic accuracy and cultural appropriateness, supporting conceptual equivalence in the local context.

A notable strength of this study is the inclusion of individuals with lived experience of mental health conditions in the expert panel. Their participation enriched the process by ensuring that item relevance and comprehensibility were evaluated not only from academic and clinical standpoints, but also from the perspective of those most affected by mental health literacy gaps. This participatory approach aligns with growing calls for co-produced research in global mental health and reinforces the importance of epistemic justice in tool development.

However, the study has limitations: The small sample size for expert content validation and pilot testing may restrict generalizability, particularly in Bolivia’s culturally and linguistically diverse regions. This underscores the potential need for additional and complete validation processes tailored to specific cultural and linguistic contexts within the country. Although multidisciplinary, the panel size (n = 8) may not capture the full range of perspectives needed for broader application.

On the other hand, the analysis of the S-CVI results—particularly the S-CVI/UA index—showed values below the recommended thresholds, indicating that further refinement of the items was needed. Nonetheless, it should be emphasized that increasing the number of evaluators makes it progressively more challenging to obtain high levels of universal agreement. However, it is important to note that the qualitative phase of this study was guided by the combined I-CVI and modified kappa results, which were deemed sufficient to inform content validity. Consequently, the main limitation is that the qualitative phase, during which experts proposed improvements to the instrument, relied exclusively on the I-CVI and modified kappa values rather than incorporating the S-CVI/UA findings.

Another limitation is the lack of quantitative verification to confirm whether the indices that informed the qualitative improvement phase actually changed, or to use such verification to establish more objective quantitative criteria for item retention.

Additionally, psychometric properties—such as internal consistency, construct validity, and test–retest reliability— were not assessed in the current phase and should be addressed in future stages of the study. Furthermore, a more in-depth analysis is needed to establish the final structure of the instrument and evaluate its reliability across the target populations. Based on the results of this subsequent analysis, it may be possible—though not guaranteed—to propose a rating scale aligned with the instrument’s finalized structure. Finally, while instruments were adapted for the urban Bolivian context, further modifications may be required to ensure suitability for rural populations, particularly with regard to different regions and local language adaptations. Ideally, such adaptations should be accompanied by a full validation process using structured samples that include groups diversified by age, sex, socioeconomic status, and educational level. In this sense, this article presents the assessment of content validity as the first stage in the overall validation process of the instrument. Further analyses will be required to examine construct validity and reliability with specific techniques and metrics.

### 4.3 Public health relevance in Bolivia and the region

Based on the findings of this study, the availability of a culturally adapted Bolivian version of a MHL assessment instrument with acceptable—although not optimal—content validity has important implications for public health at both national and regional levels. First, this instrument provides a solid empirical basis for the systematic identification of existing gaps in knowledge, attitudes, symptom recognition, and stigma related to mental health in two strategic populations: the general population and primary healthcare personnel. This dual focus is particularly relevant in contexts such as Bolivia, where primary care represents the main point of contact with the health system and where workers at this level play a fundamental mediating role between specialized services and the community. The use of this instrument would allow for the generation of comparable situational diagnoses across regions, health facilities, or population groups, thereby guiding the design of MHL programs tailored to real needs and local priorities, rather than relying on generic or imported interventions lacking contextual adaptation.

Moreover, the periodic application of this instrument would facilitate the monitoring and evaluation of MHL programs implemented at national or subnational levels, enabling the assessment of changes in key dimensions such as early recognition of mental disorders, stigma reduction, and willingness to seek professional help. This is particularly relevant in a resource-constrained country, where efficiency in budget allocation is critical and where evidence of impact can strengthen the sustainability of interventions. The availability of standardized MHL indicators would also support the integration of mental health into public health information systems, contributing to more comprehensive planning aligned with the primary healthcare approach and with international commitments, such as the achievement of Sustainable Development Goal 3.

From a public policy perspective, the use of this instrument can support the formulation of evidence-based national mental health strategies by providing local data that substantiate the need to strengthen mental health education, health workforce training, and community awareness campaigns. In a context such as Bolivia, where investment in mental health has historically been low, the generation of robust local evidence can become a key advocacy tool to make the magnitude of the problem visible and to justify increased resource allocation. Furthermore, having been developed through a rigorous process of cultural adaptation and content validation, the instrument reduces the risk of measurement bias and enhances the legitimacy of the findings among policymakers, health professionals, and other key stakeholders.

Finally, this instrument not only has potential for use within Bolivia but, together with other experiences in the region, may serve as a reference for other Latin American countries with similar sociocultural and structural characteristics. Its comparative application could contribute to the generation of regional evidence on MHL, fostering the exchange of best practices and the development of more coherent and contextually grounded mental health policies across the region. Overall, the Bolivian version of the MHL instrument represents a strategic tool for strengthening evidence-based decision-making, improving the effectiveness of mental health promotion and prevention programs, and advancing toward more equitable, culturally appropriate, and responsive health systems.

## 5 Conclusion

This study reports the translation, cultural adaptation, and content validation of a MHL assessment instrument in Bolivia. A mixed-methods design, integrating quantitative analysis and expert interviews, ensured the tool is linguistically accurate and culturally appropriate for the local context. The resulting instrument demonstrates acceptable content validity in the Bolivian context following a systematic translation and cultural adaptation process, and effectively addresses key MHL dimensions, including mental health knowledge, symptom recognition, and stigma.

## Data Availability

The raw data supporting the conclusions of this article will be made available by the authors, without undue reservation.
