# Supplementary figures and images for: Translation, cultural adaptation, and content validity evaluation of a mental health literacy instrument in Bolivia
Source: Front Public Health. 2026 Feb 25;14:1685333. doi: 10.3389/fpubh.2026.1685333 (PMC12975736; doi:10.3389/fpubh.2026.1685333)

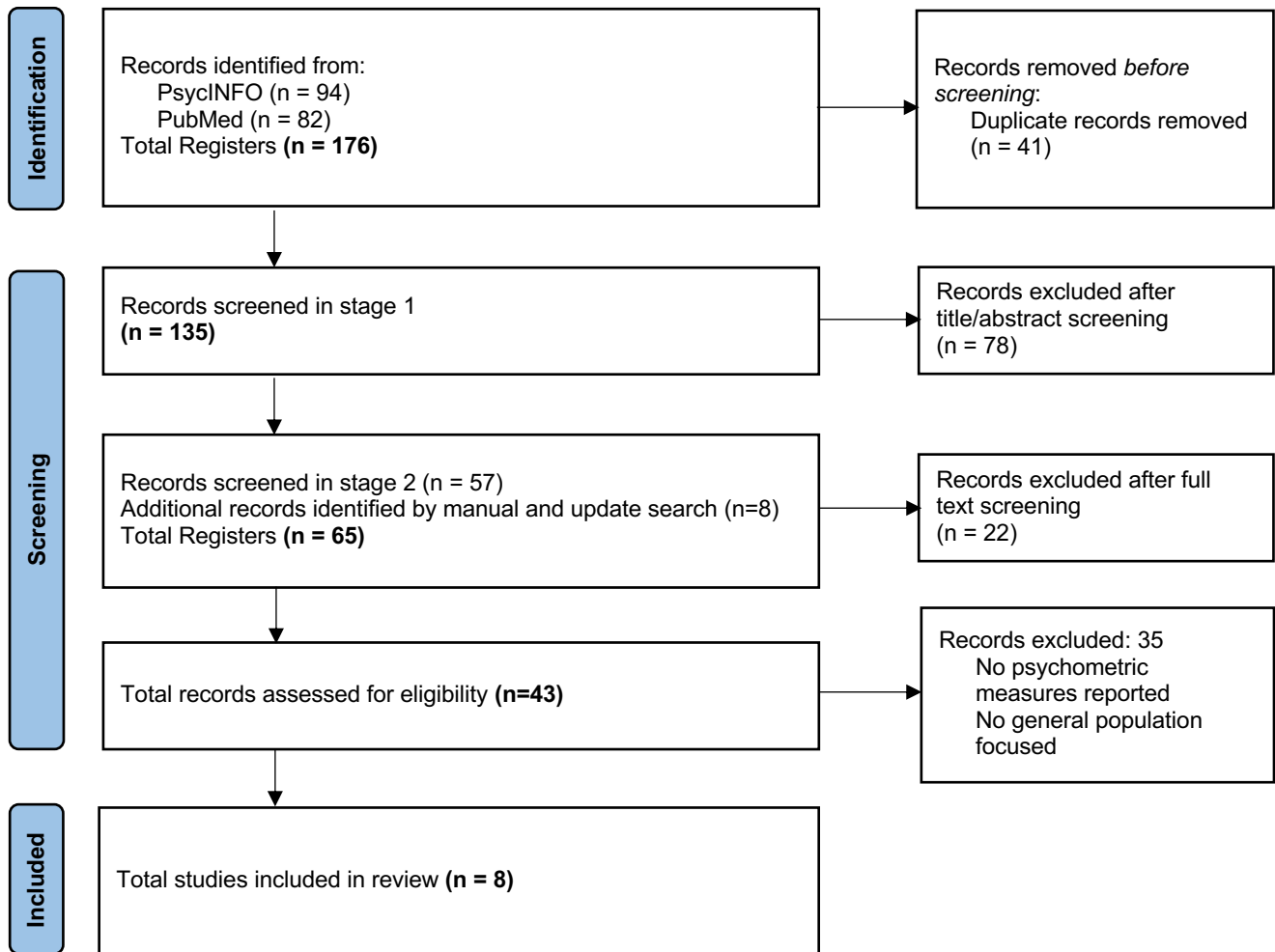

PRISMA flow diagram study selection

Supplement: Supplementary file 1 [file Table_1.pdf]
