# Supplementary material for: Translation, cultural adaptation, and content validity evaluation of a mental health literacy instrument in Bolivia
Source: Front Public Health. 2026 Feb 25;14:1685333. doi: 10.3389/fpubh.2026.1685333 (PMC12975736; doi:10.3389/fpubh.2026.1685333)
Supplement: Supplementary file 2 [file Table_2.pdf]

Supplementary material 2: Content-validity assessment protocol

Step 1: Selection of the expert panel

Experts should be selected based on their specialized knowledge in relevant fields and/or specific professional or experiential backgrounds. The panel should consist of:

a) Two health professionals specialized in clinical mental health care;

b) Two health professionals with experience in health research and validation of mental health assessment instruments;

c) Two health professionals with experience in primary-level care, including the management of mental health problems;

d) Two experiential experts, defined as individuals with personal or family experience as patients affected by mental health problems.

To qualify as an expert, participants should have a minimum of five years of experience in one of the domains described above.

Step 2: Orientation of experts to the content validation process

Following recruitment and completion of informed consent, each expert should participate in an individual online meeting. During this meeting, the following elements should be reviewed and explained:

a) The purpose of the assessment and the conceptual framework guiding it;

b) A detailed description and analysis of the instrument being evaluated;

c) The structure and scoring procedures of the quantitative content validity response form;

d) The guiding questions for the qualitative interview aimed at improving the instrument.

Step 3: Collection of quantitative data from experts

The following procedures should be implemented to collect quantitative data:

a) Each expert should receive a complete copy of the instrument and the evaluation form to be completed;

b) Experts should rate each item across three dimensions: relevance, comprehensiveness, and comprehensibility;

c) Each dimension should be rated using a 4-point Likert scale (e.g., for relevance: 1 = not relevant, 2 = somewhat relevant, 3 = quite relevant, 4 = highly relevant);

d) All completed forms should be submitted by a designated deadline.

Step 4: Analysis of Quantitative Data

After collecting the completed evaluation forms, a database should be created to conduct the corresponding analyses of the content validity indices. The following procedures should be followed:

a) Create and manage the database using software such as Microsoft Excel and SPSS;

b) Generate the Item-Level Content Validity Index (I-CVI), which represents the proportion of experts rating an item as 3 or 4, indicating agreement regarding the relevance, comprehensiveness, and comprehensibility of each item. For a panel of 8 experts, I-CVI values ≥ 0.78 should be considered acceptable;

c) Generate the modified Kappa statistic for each item to adjust I-CVI values for chance agreement and better assess expert consensus across the three evaluated dimensions. Interpretation thresholds should follow established criteria: poor (<0.4), fair (0.4–0.59), good (0.6–0.74), and excellent (≥0.75);

d) Items with scores below the defined "optimal" thresholds (I-CVI < 0.78 or modified Kappa < 0.75) should be flagged for further analysis in the qualitative phase.

Step 5: Conducting and Analyzing Qualitative Interviews

a) Semi-structured Interview Guide:

A semi-structured guide should be used to explore experts' perceptions of each item and the instrument as a whole. The theoretical framework of the three COSMIN measurement properties (relevance, comprehensiveness, and comprehensibility) and their characteristics should guide the coding approach, ensuring systematic exploration of item clarity, wording, conceptual fit, and potential improvements.

b) Item-by-Item Feedback:

- Experts should comment on items with suboptimal I-CVI and modified Kappa scores, as well as any items perceived as ambiguous, redundant, problematic, or missing;
- Experts should evaluate whether the instrument adequately covers all relevant dimensions of the construct, recommending additions or revisions as needed;

- Experts should assess linguistic clarity, reading difficulty, and cultural appropriateness of the items, noting any terms or expressions that may be misunderstood by the target population;
- Interviews should be audio-recorded (with consent), and notes should be taken to ensure accurate capture of expert feedback.

c) Qualitative Data Analysis:

A thematic content analysis should be conducted to identify recurring themes, item-level concerns, and recommendations. Coding and theme development should be iterative and compared across experts to ensure consistency.

d) Synthesis of Findings:

Qualitative insights should guide decisions on item modification, deletion, or addition, prioritizing items with low I-CVI/modified Kappa values and those flagged by multiple experts.

e) Conciliation Review:

A research experts panel composed of researchers with experience in instrument development and validation should define required changes, provide rationale, and propose revised wording or restructuring of items based on the findings.

Step 6: Piloting the Instrument

The revised instrument, incorporating feedback from both the quantitative and qualitative phases, should be pilot-tested with a sample from the target population.

- This pilot should assess comprehensiveness, comprehensibility, and usability.
- Cognitive interviews should be conducted to gather participants' feedback on item clarity, ease of completion, and overall understanding of the instrument.
