# Supplementary material for: Translation, cultural adaptation, and content validity evaluation of a mental health literacy instrument in Bolivia
Source: Front Public Health. 2026 Feb 25;14:1685333. doi: 10.3389/fpubh.2026.1685333 (PMC12975736; doi:10.3389/fpubh.2026.1685333)
Supplement: Supplementary file 3 [file Table_3.pdf]

Supplementary material 3: Qualitative interview guide

Relevance

1. Are the included questions relevant to the construct of interest?
2. What modifications would be necessary to improve the relevance of the question?
3. Are the included questions relevant for the target population?
4. Are the included questions relevant for the context of interest?
5. Are the response options appropriate?
6. Is the recall period appropriate?

Comprehensiveness

7. Are there any key concepts related to the construct that are missing?
8. Do the questions, as a whole, adequately represent the construct or dimension?

Comprehensibility

9. Will the instructions be understandable to the population of interest?
10. Will the population of interest understand the items and response options?
11. Are the items appropriately worded?
12. Do the response options match the question?
