# Supplementary material for: Translation, cultural adaptation, and content validity evaluation of a mental health literacy instrument in Bolivia
Source: Front Public Health. 2026 Feb 25;14:1685333. doi: 10.3389/fpubh.2026.1685333 (PMC12975736; doi:10.3389/fpubh.2026.1685333)
Supplement: Supplementary file 5 [file Table_5.docx]

**Supplementary material 5**

**Table 5. Constructs and tests items´ content validity assessment, dimension: Comprehensiveness**

| **Source instrument** | **Construct / Item number** | **Number of experts in agreement †** | **ICV-I ^a^** | **ICV-I interpretation** | **Pc ^b^** | **k* ^c^** | **k* interpretation** | **S-CVI/UA ^d^** | **S-CVI/Ave ^e^** |
| --- | --- | --- | --- | --- | --- | --- | --- | --- | --- |
| **MHLS - Mental Health Literacy Scale**^16^ | **Recognition of disorders** | | | | | | | 0.00 | 0.81 |
|  | 1 | 7 | 0.88 | Acceptable | 0.03 | 0.87 | Excellent |  |  |
|  | 2 | 7 | 0.88 | Acceptable | 0.03 | 0.87 | Excellent |  |  |
|  | 3 | 7 | 0.88 | Acceptable | 0.03 | 0.87 | Excellent |  |  |
|  | **4** | 5 | 0.63 | For review | 0.22 | 0.52 | Fair |  |  |
|  | **5** | 6 | 0.75 | For review | 0.11 | 0.72 | Good |  |  |
|  | **6** | 6 | 0.75 | For review | 0.11 | 0.72 | Good |  |  |
|  | 7 | 7 | 0.88 | Acceptable | 0.03 | 0.87 | Excellent |  |  |
|  | 8 | 7 | 0.88 | Acceptable | 0.03 | 0.87 | Excellent |  |  |
|  | **Knowledge of risk factors and causes** | | | | | | | 0.00 | 0.75 |
|  | **9** | 6 | 0.75 | For review | 0.11 | 0.72 | Good |  |  |
|  | **10** | 6 | 0.75 | For review | 0.11 | 0.72 | Good |  |  |
|  | **Self-treatment knowledge** | | | | | | | 0.00 | 0.63 |
|  | **11** | 5 | 0.63 | For review | 0.22 | 0.52 | Fair |  |  |
|  | **12** | 5 | 0.63 | For review | 0.22 | 0.52 | Fair |  |  |
|  | **Knowledge of professional help available** | | | | | | | 0.00 | 0.67 |
|  | **13** | 6 | 0.75 | For review | 0.11 | 0.72 | Good |  |  |
|  | **14** | 5 | 0.63 | For review | 0.22 | 0.52 | Fair |  |  |
|  | **15** | 5 | 0.63 | For review | 0.22 | 0.52 | Fair |  |  |
|  | **Knowledge of how to seek mental health information** | | | | | | | 0.75 | 0.97 |
|  | 16 | 8 | 1.00 | Acceptable | 0.00 | 1.00 | Excellent |  |  |
|  | 17 | 8 | 1.00 | Acceptable | 0.00 | 1.00 | Excellent |  |  |
|  | 18 | 8 | 1.00 | Acceptable | 0.00 | 1.00 | Excellent |  |  |
|  | 19 | 7 | 0.88 | Acceptable | 0.03 | 0.87 | Excellent |  |  |
|  | **Attitudes that promote recognition and appropriate help-seeking** | | | | | | | 0.31 | 0.82 |
|  | **20** | 5 | 0.63 | For review | 0.22 | 0.52 | Fair |  |  |
|  | **21** | 5 | 0.63 | For review | 0.22 | 0.52 | Fair |  |  |
|  | **22** | 5 | 0.63 | For review | 0.22 | 0.52 | Fair |  |  |
|  | **23** | 6 | 0.75 | For review | 0.11 | 0.72 | Good |  |  |
|  | **24** | 5 | 0.63 | For review | 0.22 | 0.52 | Fair |  |  |
|  | 25 | 7 | 0.88 | Acceptable | 0.03 | 0.87 | Excellent |  |  |
|  | **26** | 6 | 0.75 | For review | 0.11 | 0.72 | Good |  |  |
|  | **27** | 6 | 0.75 | For review | 0.11 | 0.72 | Good |  |  |
|  | 28 | 7 | 0.88 | Acceptable | 0.03 | 0.87 | Excellent |  |  |
|  | 29 | 8 | 1.00 | Acceptable | 0.00 | 1.00 | Excellent |  |  |
|  | 30 | 7 | 0.88 | Acceptable | 0.03 | 0.87 | Excellent |  |  |
|  | 31 | 8 | 1.00 | Acceptable | 0.00 | 1.00 | Excellent |  |  |
|  | 32 | 8 | 1.00 | Acceptable | 0.00 | 1.00 | Excellent |  |  |
|  | 33 | 8 | 1.00 | Acceptable | 0.00 | 1.00 | Excellent |  |  |
|  | **34** | 6 | 0.75 | For review | 0.11 | 0.72 | Good |  |  |
|  | 35 | 8 | 1.00 | Acceptable | 0.00 | 1.00 | Excellent |  |  |
| **PDDS - Perceived discrimination and devaluation scale**^29^ | **Self-reported perceived discrimination and devaluation towards mental illness** | | | | | | | 0.45 | 0.91 |
|  | **36** | 6 | 0.75 | For review | 0.11 | 0.72 | Good |  |  |
|  | 37 | 8 | 1.00 | Acceptable | 0.00 | 1.00 | Excellent |  |  |
|  | 38 | 7 | 0.88 | Acceptable | 0.03 | 0.87 | Excellent |  |  |
|  | **39** | 6 | 0.75 | For review | 0.11 | 0.72 | Good |  |  |
|  | 40 | 8 | 1.00 | Acceptable | 0.00 | 1.00 | Excellent |  |  |
|  | 41 | 8 | 1.00 | Acceptable | 0.00 | 1.00 | Excellent |  |  |
|  | 42 | 7 | 0.88 | Acceptable | 0.03 | 0.87 | Excellent |  |  |
|  | 43 | 7 | 0.88 | Acceptable | 0.03 | 0.87 | Excellent |  |  |
|  | 44 | 7 | 0.88 | Acceptable | 0.03 | 0.87 | Excellent |  |  |
|  | 45 | 7 | 0.88 | Acceptable | 0.03 | 0.87 | Excellent |  |  |
|  | 46 | 8 | 1.00 | Acceptable | 0.00 | 1.00 | Excellent |  |  |
|  | 47 | 8 | 1.00 | Acceptable | 0.00 | 1.00 | Excellent |  |  |
| **MHPR - Mental health problem recognition module**^30^ | **Recognition of disorders symptoms** | | | | | | | 0.00 | 0.88 |
|  | 48 | 7 | 0.88 | Acceptable | 0.03 | 0.87 | Excellent |  |  |
|  | 49 | 7 | 0.88 | Acceptable | 0.03 | 0.87 | Excellent |  |  |
|  | 50 | 7 | 0.88 | Acceptable | 0.03 | 0.87 | Excellent |  |  |
|  | 51 | 7 | 0.88 | Acceptable | 0.03 | 0.87 | Excellent |  |  |
|  | 52 | 7 | 0.88 | Acceptable | 0.03 | 0.87 | Excellent |  |  |
|  | 53 | 7 | 0.88 | Acceptable | 0.03 | 0.87 | Excellent |  |  |

All items were rated by N=8 experts.

Items scoring below optimal thresholds (I-CVI ≥ 0.78; k > 0.74) appear in bold.

**†**The number of raters who agreed on ratings of 3 or 4 on the Likert scale is shown.

^a^ I-CVI (item content validity index): number of experts giving a rating of 3 or 4/number of experts. Evaluation criteria: Acceptable = ≥ 0.78; for review = < 0.78.

^b^ Pc (probability of a chance occurrence) = [N!/A!(N - A)!] x 0.5N, where N = number of experts and A = number of agreeing experts

^c^ k* = kappa designating agreement on dimension: k* = (I-CVI – Pc)/(1 – Pc). Evaluation criteria: Poor = k* < 0.40; fair = k* of 0.40–0.59; good = k* of 0.60–0.74; and excellent = k* > 0.74.

^d^ S-CVI/UA (Universal Agreement among experts): Recommended value ≥ 0.80 indicative of acceptable content validity.

^e^ S-CVI/Ave (Average content validity index): Recommended value ≥ 0.90 indicative of excellent content validity.
