# Supplementary material for: Translation, cultural adaptation, and content validity evaluation of a mental health literacy instrument in Bolivia
Source: Front Public Health. 2026 Feb 25;14:1685333. doi: 10.3389/fpubh.2026.1685333 (PMC12975736; doi:10.3389/fpubh.2026.1685333)
Supplement: Supplementary file 7 [file Table_7.docx]

**Supplementary material 7**

**Ítems del instrumento de evaluación de alfabetización en salud mental**

**Reconocimiento de Problemas de Salud Mental**

|  | **Muy improbable** | **Improbable** | **Probable** | **Muy probable** |
| --- | --- | --- | --- | --- |
| 1. Si alguien se pusiera demasiado nervioso o ansioso en situaciones con otras personas (por ejemplo, una fiesta) o situaciones en las que debe hablar (por ejemplo, una exposición en una reunión), en las que tiene miedo de ser evaluado por otros y sentirse humillado o avergonzado, entonces ¿Qué tan probable es que tenga **Fobia social**? |  |  |  |  |
| 2. Si alguien tuvo una preocupación muy grande por una serie de eventos o actividades en los que ese nivel de preocupación no estaba justificado, y tuvo dificultades para controlar esta preocupación, presentando síntomas físicos como músculos tensos y sensación de cansancio, ¿Qué tan probable es que tenga **Trastorno de ansiedad generalizada**? |  |  |  |  |
| 3. Si alguien estuvo decaído durante dos o más semanas, perdió el placer o el interés en sus actividades normales, y experimentó cambios en el apetito y el sueño, ¿Qué tan probable es que tenga un **Trastorno depresivo mayor**? |  |  |  |  |
| 4. ¿Qué tan probable es que los **Trastornos de personalidad** sean un tipo de enfermedad mental? |  |  |  |  |
| 5. ¿Qué tan probable es que la **Distimia** sea un problema de salud mental? |  |  |  |  |
| 6. ¿Qué tan probable es que el diagnóstico de **Agorafobia** incluya ansiedad ante situaciones en las que escapar puede ser difícil o vergonzoso? |  |  |  |  |
| 7. ¿Qué tan probable es que el diagnóstico de **Trastorno bipolar** incluya experimentar períodos de estado de ánimo eufórico (es decir, alto) y períodos de estado de ánimo deprimido (es decir, bajo)? |  |  |  |  |
| 8. ¿Qué tan probable es que el diagnóstico de **Drogodependencia** incluya la resistencia física y psicológica a la droga (es decir, necesitar más droga para obtener el mismo efecto)? |  |  |  |  |

**Conocimiento de Factores de Riesgo y Causas**

|  | **Muy improbable** | **Improbable** | **Probable** | **Muy probable** |
| --- | --- | --- | --- | --- |
| 9. ¿Qué tan probable es que, en Bolivia las mujeres tengan MÁS probabilidades de experimentar una **enfermedad mental** de cualquier tipo, en comparación con los hombres? |  |  |  |  |
| 10. ¿Qué tan probable es que, en Bolivia, los hombres tengan MÁS probabilidades de experimentar un **trastorno de ansiedad** en comparación con las mujeres? * |  |  |  |  |

***** Indica que la puntuación del ítem señalado está invertida.

**Conocimiento de Autotratamiento**

|  | **Muy inútil** | **Inútil** | **Útil** | **Muy útil** |
| --- | --- | --- | --- | --- |
| 11. ¿Qué tan útil sería para alguien que tuviera dificultades para manejar sus emociones, mejorar su calidad de sueño (por ejemplo, si está muy ansioso o deprimido)? |  |  |  |  |
| 12. ¿Qué tan útil sería para alguien que tuviera dificultades para manejar sus emociones, evitar todas las actividades o situaciones que le hicieran sentir ansioso? * |  |  |  |  |

***** Indica que la puntuación del ítem señalado está invertida.

**Conocimiento de Ayuda Profesional Disponible**

|  | **Muy improbable** | **Improbable** | **Probable** | **Muy probable** |
| --- | --- | --- | --- | --- |
| 13. ¿Qué tan probable es que la **Terapia Cognitivo Conductual (TCC)** sea una terapia basada en desafiar los pensamientos negativos y aumentar las conductas útiles? |  |  |  |  |
| Los profesionales de la salud mental están obligados a respetar la confidencialidad; sin embargo, hay situaciones en las que esto no se aplica. |  |  |  |  |
| 14 ¿Qué tan probable es, que a un profesional de la salud mental se le permita romper la confidencialidad en la siguiente situación?: Si el profesional considera que usted (como paciente) corre riesgo inmediato de hacerse daño a sí mismo o a otras personas |  |  |  |  |
| 15. ¿Qué tan probable es, que a un profesional de la salud mental se le permita romper la confidencialidad en la siguiente situación?: Si el profesional considera que usted (como paciente) tiene un problema que no pone en peligro su vida, pero quiere avisarles a otras personas de ese problema para que ellos le brinden un mejor apoyo * |  |  |  |  |

***** Indica que la puntuación del ítem señalado está invertida.

**Conocimiento de Cómo Buscar Información sobre Salud Mental**

|  | **Totalmente en desacuerdo** | **En desacuerdo** | **Ni en desacuerdo, ni de acuerdo** | **De acuerdo** | **Totalmente de acuerdo** |
| --- | --- | --- | --- | --- | --- |
| 16. Me siento confiado de saber dónde buscar información sobre enfermedades mentales. |  |  |  |  |  |
| 17. Me siento confiado de poder utilizar la computadora o el teléfono para buscar información sobre enfermedades mentales. |  |  |  |  |  |
| 18. Me siento confiado para asistir a consultas presenciales buscando información sobre enfermedades mentales (por ejemplo, consultar al médico general). |  |  |  |  |  |
| 19. Me siento confiado de disponer de personas y recursos (p. ej., médico general, Internet, amigos) con los que puedo buscar información sobre enfermedades mentales. |  |  |  |  |  |

**Actitudes que Promueven el Reconocimiento y la Búsqueda de Ayuda Adecuada**

|  | **Totalmente en desacuerdo** | **En desacuerdo** | **Ni en desacuerdo, ni de acuerdo** | **De acuerdo** | **Totalmente de acuerdo** |
| --- | --- | --- | --- | --- | --- |
| 20. Las personas con una enfermedad mental podrían recuperarse si quisieran. * |  |  |  |  |  |
| 21. Una enfermedad mental es un signo de debilidad personal. * |  |  |  |  |  |
| 22. Una enfermedad mental no es una enfermedad médica real. * |  |  |  |  |  |
| 23. Las personas con una enfermedad mental son peligrosas. * |  |  |  |  |  |
| 24. Es mejor evitar a las personas con una enfermedad mental para no desarrollar este problema. * |  |  |  |  |  |
| 25. Si yo tuviera una enfermedad mental no se lo diría a nadie. * |  |  |  |  |  |
| 26. Ver a un profesional de la salud mental significa que no eres lo bastante fuerte para manejar tus propias dificultades. * |  |  |  |  |  |
| 27. Si tuviera una enfermedad mental, no buscaría ayuda de un profesional de la salud mental. * |  |  |  |  |  |
| 28. Creo que el tratamiento para una enfermedad mental, proporcionado por un profesional de la salud mental, no sería efectivo. * |  |  |  |  |  |

* Indica que la puntuación del ítem señalado está invertida.

|  | **Definitivamente no dispuesto** | **Probablemente no dispuesto** | **Ni dispuesto, Ni no dispuesto** | **Probablemente dispuesto** | **Definitivamente dispuesto** |
| --- | --- | --- | --- | --- | --- |
| 29. ¿Qué tan dispuesto estaría a mudarse al lado de alguien con una enfermedad mental? |  |  |  |  |  |
| 30. ¿Qué tan dispuesto estaría a pasar una noche socializando con alguien que tiene una enfermedad mental? |  |  |  |  |  |
| 31. ¿Qué tan dispuesto estaría a hacerse amigo de alguien con una enfermedad mental? |  |  |  |  |  |
| 32. ¿Qué tan dispuesto estaría a que alguien con una enfermedad mental comenzara a trabajar cercanamente con usted? |  |  |  |  |  |
| 33. ¿Qué tan dispuesto estaría a que alguien con una enfermedad mental se casara con un miembro de su familia? |  |  |  |  |  |
| 34. ¿Qué tan dispuesto estaría a votar por un político si supiera que ha sufrido una enfermedad mental? |  |  |  |  |  |
| 35. ¿Qué tan dispuesto estaría a contratar a alguien si supiera que tiene una enfermedad mental? |  |  |  |  |  |

**Auto-reporte de Discriminación y Desvalorización Percibida hacia las Enfermedades Mentales**

|  | **Totalmente en desacuerdo** | **En desacuerdo** | **De acuerdo** | **Totalmente de acuerdo** |
| --- | --- | --- | --- | --- |
| 36. La mayoría de la gente aceptaría de buena gana a una persona que tuvo un problema de salud mental como amigo cercano. * |  |  |  |  |
| 37. La mayoría de la gente cree que una persona que ha estado en un hospital psiquiátrico es tan inteligente como la persona promedio. |  |  |  |  |
| 38. La mayoría de la gente cree que una persona que tuvo un problema de salud mental es tan digna de confianza como el ciudadano promedio. * |  |  |  |  |
| 39. La mayoría de la gente aceptaría a una persona que tuvo un problema de salud completamente recuperada, como maestro de niños pequeños en una escuela pública. * |  |  |  |  |
| 40. La mayoría de las personas sienten que ingresar a un hospital psiquiátrico es una señal de fracaso personal. |  |  |  |  |
| 41. La mayoría de la gente no contrataría a una persona que tuvo un problema de salud mental para que cuidara de sus hijos, incluso si hubiera estado bien durante algún tiempo. |  |  |  |  |
| 42. La mayoría de la gente menosprecia a una persona que ha estado en un hospital psiquiátrico. * |  |  |  |  |
| 43. La mayoría de los empleadores contratarán a una persona que tuvo un problema de salud mental si está calificado para el trabajo. * |  |  |  |  |
| 44. La mayoría de los empleadores pasarán por alto la solicitud de una persona que tuvo un problema de salud mental en favor de otro solicitante |  |  |  |  |
| 45. La mayoría de las personas en mi comunidad tratarían a una persona que tuvo un problema de salud mental tal como tratarían a cualquier persona. |  |  |  |  |
| 46. La mayoría de las mujeres jóvenes se mostrarían reacias a salir con un hombre que ha sido hospitalizado por un trastorno mental grave. * |  |  |  |  |
| 47. La mayoría de las personas, una vez que saben que una persona estuvo en un hospital psiquiátrico, toman sus opiniones menos en serio. |  |  |  |  |

* Indica que la puntuación del ítem señalado está invertida.

**Reconocimiento de Síntomas de Problemas de Salud Mental**

48. Seleccione **2 opciones** que le harían pensar que una persona tiene **Depresión**. Si no sabe, por favor marque la alternativa “No sé”.

a) Estar más triste

b) Pérdidas de memoria

c) Escuchar voces que otros no oyen

d) Haber bajado mucho de peso en corto tiempo

e) Pérdida del interés

f) No poder dejar las bebidas alcohólicas

g) No sé

49. Seleccione **2 opciones** que le harían pensar que una persona está teniendo un episodio de **Psicosis como, esquizofrenia**. Si no sabe, por favor marque la alternativa “No sé”.

a) Pérdidas de memoria

b) Escuchar voces que otros no oyen

c) Tener ideas extrañas (ideas delirantes/persecutorias), que otras personas no comparten

d) Haber subido mucho de peso

e) Consumir drogas ilícitas en exceso

f) Estar más triste que lo habitual

g) No sé

50. Seleccione **2 opciones** que le harían pensar que una persona es **Alcohólica** o tiene problemas por el consumo de alcohol. Si no sabe, por favor marque la alternativa No sé.

a) Tener ideas extrañas (ideas delirantes/persecutorias), que otras personas no comparten

b) Tiene pérdidas de memoria u olvidos que son frecuentes

c) Cuando bebe alcohol le cuesta parar de tomar y sigue hasta quedar borracho

d) Tener crisis de angustia muy intensas, sin relación con el consumo de alcohol

e) Se siente muy triste y no tiene interés en hacer sus tareas habituales

f) Ha tenido problemas con su familia o con el trabajo, por la forma en que bebe alcohol

g) No sé

51. Seleccione **2 opciones** que le harían pensar que una persona tiene un problema de **Ansiedad**. Si no sabe, por favor marque la alternativa No sé.

a) Tener una preocupación excesiva por las cosas que a uno le suceden

b) Estar más triste

c) Pérdidas de memoria

d) La persona parece siempre inquieta y nerviosa

e) Sentir fatiga o poca energía

f) No comer bien y haber subido mucho de peso

g) No sé

52. Seleccione **2 opciones** que le harían pensar que una persona tiene **Trastorno de la personalidad**. Si no sabe, por favor marque la alternativa No sé.

a) Tiene pérdidas de memoria u olvidos que son frecuentes

b) Inestabilidad emocional

c) Tener pensamientos desordenados

d) Escuchar voces que otros no oyen

e) Dificultad de establecer relaciones interpersonales duraderas y estables

f) Sentir fatiga o poca energía

g) No sé

53. Seleccione **2 opciones** que le harían pensar que una persona está pensando en matarse o que tiene **ideas suicidas**. Si no sabe, por favor marque la alternativa No sé.

a) Tener una preocupación excesiva por todas las cosas que pasan

b) Estar triste y desanimado

c) Hablar de muerte como un alivio de los problemas

d) La persona parece siempre inquieta y nerviosa

e) Decir que ha pensado en matarse para aliviar su pesar

f) Estar con poco apetito y haber perdido peso en las últimas semanas

g) Alejarse/ aislarse de otras personas

h) No sé
